# Acid-Base Disorders in COVID-19 Patients with Acute Respiratory Distress Syndrome

**DOI:** 10.3390/jcm11082093

**Published:** 2022-04-08

**Authors:** Davide Chiumello, Tommaso Pozzi, Isabella Fratti, Leo Modafferi, Marialaura Montante, Giuseppe Francesco Sferrazza Papa, Silvia Coppola

**Affiliations:** 1 Department of Anesthesia and Intensive Care, ASST Santi Paolo e Carlo, San Paolo University Hospital, Via Di Rudini 9, 20122 Milan, Italy; silvia_coppola@libero.it; 2 Department of Health Sciences, University of Milan, 20122 Milan, Italy; tommaso.pozzi@unimi.it (T.P.); isabella.fratti@unimi.it (I.F.); leo.modafferi@unimi.it (L.M.); marialaura.montante@unimi.it (M.M.); giuseppe.sferrazza@unimi.it (G.F.S.P.); 3 Coordinated Research Center on Respiratory Failure, University of Milan, 20122 Milan, Italy; 4 Department of Neurorehabilitation Sciences, Casa di Cura del Policlinico, 20144 Milan, Italy

**Keywords:** COVID-19, ARDS, acid-base disorders, non-invasive ventilation

## Abstract

Our aim was to investigate the distribution of acid-base disorders in patients with COVID-19 ARDS using both the Henderson–Hasselbalch and Stewart’s approach and to explore if hypoxemia can influence acid-base disorders. COVID-19 ARDS patients, within the first 48 h of the need for a non-invasive respiratory support, were retrospectively enrolled. Respiratory support was provided by helmet continuous positive airway pressure (CPAP) or by non-invasive ventilation. One hundred and four patients were enrolled, 84% treated with CPAP and 16% with non-invasive ventilation. Using the Henderson–Hasselbalch approach, 40% and 32% of patients presented respiratory and metabolic alkalosis, respectively; 13% did not present acid-base disorders. Using Stewart’s approach, 43% and 33% had a respiratory and metabolic alkalosis, respectively; 12% of patients had a mixed disorder characterized by normal pH with a lower SID. The severe hypoxemic and moderate hypoxemic group presented similar frequencies of respiratory and metabolic alkalosis. The most frequent acid-base disorders were respiratory and metabolic alkalosis using both the Henderson–Hasselbalch and Stewart’s approach. Stewart’s approach detected mixed disorders with a normal pH probably generated by the combined effect of strong ions and weak acids. The impairment of oxygenation did not affect acid-base disorders.

## 1. Introduction

The severe acute respiratory syndrome coronavirus 2 (SARS-CoV-2), due to the high tropism of the virus not only for the respiratory tract, but also for the bowel, heart, kidney and nervous system, can result in a high spectrum of disorders such as acute respiratory failure, acute heart failure, acute kidney injury, coagulopathy, extensive microvascular thrombosis, a dysregulated inflammatory response, sepsis and multiorgan failure [1,2,3,4]. As reported by Huang et al., patients with COVID-19 presented ARDS in 29%, acute kidney injury in 13% and cardiac failure in 12% of the cases [5].

In the presence of an acute respiratory failure, 81% of the patients required oxygen support and, based on the severity of acute respiratory failure (i.e., level of hypoxemia and dyspnea), most of these patients also required a respiratory support. The main applied respiratory supports included high flow oxygen therapy, continuous positive airway pressure (CPAP), non-invasive (NIV) and invasive mechanical ventilation. The proportion of patients treated with non-invasive respiratory supports varied from 62% in China to 20% and 11% in North America and Italy, respectively [1,6,7]. 

The presence of an altered organ function (lung, heart and kidney) influencing the patient homeostasis could induce an alteration in the acid-base balance according to the severity of the underlying disease [8]. However, limited data have been available to describe the acid-base characteristics of COVID-19 patients in the early phase of hospital admission. In more than one thousand critically ill patients, the alteration in pH at admission was significantly associated with the mortality [7]. Alfano et al., in a retrospective study of COVID-19 patients receiving oxygen therapy, found acid-base disturbances in 80% of the patients and the main alterations were the metabolic and respiratory alkalosis [9]. Patients with respiratory alkalosis had higher ratios of underlying disease and were more likely to die compared to patients without respiratory alkalosis [10].

The traditional classification of the acid-base disorders, in terms of presence of alkalemia or acidemia due to a respiratory versus metabolic disorder, has been based on the Henderson–Hasselbalch equation, which focuses on the plasma bicarbonate concentration ([HCO3^−^]), plasma carbon dioxide tension (PCO_2_) and the negative logarithm of the apparent dissociation constant (p*K*_1_′) for carbonic acid (H_2_CO_3_) in plasma [11]. Although this approach is the most widely used to identify an acid-base derangement, it is merely descriptive rather than mechanistic in nature and often unable to provide a diagnosis in critically ill patients [12]. Thus, in the late 1970s a mathematical model based on physicochemical principles was proposed by Peter Stewart to describe the alterations in acid-base balance according to three different variables: the strong ion difference (SID), carbon dioxide and weak acids [13,14]. In non-COVID-19 critically ill patients, Stewart’s approach showed, compared to the traditional evaluation, a greater identification of acid-base disorders [15,16,17].

Our aim was: (1) to investigate the distribution of acid-base disorders in a cohort of patients with acute respiratory distress syndrome due to COVID-19 using both the Henderson–Hasselbalch “physiological” approach and the Stewart “physicochemical” approach within the first 48 h of the need for a non-invasive respiratory support, and (2) to explore if the hypoxemia, as marker of the severity of the disease, can influence the acid-base disorders.

## 2. Materials and Methods

### 2.1. Study Population

Adults (>18 years) with acute respiratory failure caused by COVID-19 pneumonia, PaO_2_/FiO_2_ < 300, with ground glass bilateral opacities at chest X-ray or lung CT and requirement for non-invasive respiratory support, were retrospectively enrolled. They were admitted at the intermediate-high Dependency Unit of the ASST Santi Paolo e Carlo, San Paolo Hospital, Milan between September 2020 and March 2021 and treated with non-invasive respiratory support. Exclusion criteria were: the need for immediate endotracheal intubation (ETI) and Glasgow Coma Scale < 15.

The study was approved by the local ethical board (Comitato Etico Milano Area I; 17263/2020-2020/ST/095), and informed consent was acquired from each patient.

### 2.2. Study Protocol and Data Collection

Upon their emergency department admission demographics, comorbidities and chronic therapies were recorded at admission.

Respiratory support was provided by helmet continuous positive airway pressure (CPAP) or by mask delivered non-invasive ventilation (NIV) at the discretion of the attending physician to maintain the peripheral oxygen saturation (SpO2) > 92% and a respiratory rate < 25 bpm.

After the transfer to the High Dependency, vital signs, Borg scale dyspnea score and Work Of Breathing (WOB) score [18], laboratory parameters, non-invasive respiratory support settings, respiratory rate and arterial blood gas analysis within the first 48 hours from the onset of the respiratory support were collected.

The same blood sample using a Siemens RAPIDPoint 500 blood gas analyzer (Siemens HealthCare, Erlangen, Germany) was analyzed to investigate acid-base disorders using both the Henderson–Hasselbalch approach, based on bicarbonate-carbon dioxide, and the physicochemical approach (Stewart’s).

According to the Henderson–Hasselbalch approach, firstly, patients were classified as acidemic, alkalemic and with no pH disorder; secondly, the Primary Acid-Base Disturbance was identified and an eventual compensatory secondary response, assessed using the Boston Rules [11].

- A pH of less than 7.38 was categorized as acidemia; a pH of more than 7.42 was categorized as alkalemia; a pH between 7.38 and 7.42, with PaCO_2_ between 38 and 42 mmHg and [HCO3-] between 22 and 26 mMol/L was categorized as no disorder;
- Respiratory acidosis pH < 7.38 and PaCO_2_ > 42 mmHg; respiratory acidosis with the secondary acute metabolic response if [HCO3^−^] is increased by 1 mMol/liter for each PaCO_2_ increase of 10 mmHg above 40 mm Hg; respiratory acidosis with the secondary chronic metabolic response if [HCO3^−^] is increased by 4–5 mMol/liter for each PaCO_2_ increase of 10 mmHg above 40 mmHg; superimposed metabolic alkalosis or acidosis may be diagnosed if the calculated [HCO3^−^] is greater or less than predicted;
- Metabolic acidosis pH < 7.38 and bicarbonate [HCO3^−^] < 22 mMol/L; metabolic acidosis with secondary respiratory response if PaCO_2_ = 1.5 × [HCO3^−^] + 8 ± 2 mmHg; superimposed respiratory acidosis or alkalosis may be diagnosed if the calculated PaCO_2_ is greater or less than predicted;
- Respiratory alkalosis pH > 7.42 and PaCO_2_ < 38 mmHg; respiratory alkalosis with the secondary acute metabolic response if is decreased by 2 mMol/L for each PaCO_2_ decrease of 10 mmHg below 40 mmHg; respiratory alkalosis with the secondary chronic metabolic response if [HCO3^−^] is decreased by 4–5 mMol/L for each PaCO_2_ decrease of 10 mmHg below 40 mmHg; superimposed metabolic alkalosis or acidosis may be diagnosed if the calculated [HCO3^−^] is greater or less than predicted;
- Metabolic alkalosis pH > 7.42 and [HCO3^−^] > 26 mMol/L; metabolic alkalosis with secondary respiratory response if PaCO_2_ = 0.7 × ([HCO3^−^] − 24) + 40 ± 2 mmHg; superimposed respiratory acidosis or alkalosis may be diagnosed if the calculated PaCO_2_ is greater or less than predicted.

We also calculated plasmatic Anion Gap as defined as: Anion Gap (mEq/L) = [Na^+^] – [Cl^−^] – [HCO3^−^].

According to the physicochemical approach (Stewart’s), we classified acidemia, alkalemia and no pH disorder based on the PCO_2_ and the electrolyte composition of blood (the apparent SID) [13]. The apparent SID was calculated as: [aSID] (mEq/L) = [Na^+^] + [K^+^] − [Cl^−^] − [Lactates^−^].

- A pH of less than 7.38 was categorized as acidemia; a pH of more than 7.42 was categorized as alkalemia; a pH between 7.38 and 7.42, with PaCO_2_ between 38 and 42 mmHg and [aSID] between 38 and 42 mEq/L was categorized as no disorder;
- Respiratory acidosis: pH < 7.38, PaCO_2_ > 42 mm Hg and [aSID] between 38 and 42 mEq/L;
- Metabolic acidosis secondary to aSID: pH < 7.38, PaCO_2_ between 38 and 42 and [aSID] < 38 mEq/L;
- Other metabolic acidosis: pH < 7.38, PaCO_2_ between 38 and 42 and [aSID] between 38 and 42 mEq/L;
- Respiratory alkalosis: pH > 7.42, PaCO_2_ < 38 mmHg and [aSID] 38–42 mEq/L;
- Metabolic alkalosis secondary to aSID: pH > 7.42, PaCO_2_ between 38 and 42 mmHg and [aSID] > 42 mEq/L;
- Other metabolic alkalosis: pH > 7.42, PaCO_2_ between 38 and 42 mmHg and [aSID] between 38 and 42 mEq/L;
- Mixed disorder pH 7.38–7.42 with PaCO_2_ > 42 and [aSID] > 42 mEq/L or PaCO_2_ < 38 and [aSID] < 38 mEq/L.

No advice was given to the physician regarding acid-base derangements treatment; however, sodium bicarbonate was not used in the patients.

Finally, we divided the whole population according to the severity of hypoxemia in terms of the median value of PaO_2_/FiO_2_.

### 2.3. Statistical Analysis

Categorical data are reported as % (number), while continuous variables are expressed as median [IQR]; normality of distribution was assessed by the Shapiro–Wilks tests. A One-Way Analysis of Variance (ANOVA) or Kruskal–Wallis test were used to assess differences among acid-base disturbance groups; Student’s *t*-test or Wilcoxon–Mann–Whitney test were used to assess differences between groups divided according to the median value of PaO_2_/FiO_2_. All analyses were performed with R Studio (R Foundation for Statistical Computing, Vienna, Austria).

## 3. Results

A total of 104 patients were enrolled in the study. The baseline characteristics at emergency department were shown in Table 1. The median age was 58 (52–64) years, 77 (73%) were males with a body mass index of 28 (25–33) kg/m^2^. Patients presented a median period of 6 (4–8) days from onset of the symptoms to the emergency department admission. At the hospital admission, all patients received oxygen therapy with a PaO_2_/FiO_2_ of 264 (204–301) and presented respiratory alkalosis with PaCO_2_ 31.8 (28.4–34.2) mmHg.

Within 48 h from the onset of the non-invasive respiratory support, 87 (84%) and 17 (16%) patients were treated with CPAP and non-invasive ventilation, respectively (Table 2). The median applied PEEP level was 8 (7.5–10) cmH_2_O, with a PaO_2_/FiO_2_ of 199 (139–246). The median pH was 7.44 (7.43–7.46), with a PaCO_2_ of 38 (35–41) mmHg and bicarbonate of 25.8 (24.1–27.4) mMol/L. The hospital mortality was 14%.

### 3.1. Acid-Base Disturbance According to Henderson–Hasselbalch Approach

Considering the different acid-base disorders, respiratory rate, applied PEEP and PaO_2_/FiO_2_ were not different among groups (Table 2).

**Figure 1:**
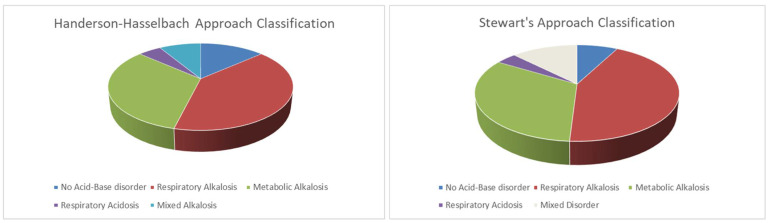
Acid-base disturbances frequencies according to Henderson–Hasselbalch (on the **left**) and Stewart’s approach (on the **right**).

Forty-two (40%) and 34 (32%) patients presented respiratory alkalosis and metabolic alkalosis, respectively (Figure 1). Patients with respiratory alkalosis had a median pH of 7.44 (7.44–7.46), with a PaCO_2_ of 35 (33–36) mmHg and bicarbonate of 24.2 (22.9–25) mMol/L. In this group of patients, the PaCO_2_ was not related to the respiratory rate nor to the hypoxemia (*p* = 0.423, R^2^ = 0.01; *p* = 0.237, R^2^ = 0.01).

Patients with metabolic alkalosis presented values of median pH of 7.45 (7.44–7.46), PaCO_2_ of 41 (40–44) mmHg and bicarbonate of 28 (27.1–29.7) mMol/L. 

Nine (10%) patients presented a mixed alkalosis due to a decreased PaCO_2_ (36 (35–37) mmHg) together with an increased bicarbonate concentration (27.0 (26.1–27.4) mMol/L).

Fourteen did not present any acid-base disorder. 

The sodium, potassium and chloride concentrations were not different among groups and creatinine levels were within normal range. No patients had a metabolic acidosis according to our criteria.

### 3.2. Acid-Base Disturbance According to Stewart’s Method

Using Stewart’s approach forty-five (43%), twenty (19%) and fourteen (14%) had a respiratory alkalosis, a metabolic alkalosis secondary to aSID and other metabolic alkalosis, respectively (Table 3, Figure 1). Comparing these three groups, the pH was similar while the PaCO_2_ was, as expected, lower in the respiratory alkalosis, while the SID was slightly higher, even if within the normal range in the metabolic alkalosis group and slightly lower in the respiratory alkalosis group. The serum creatinine was not different between these groups.

We did not find patients with superimposed respiratory and metabolic alkalosis considering the apparent SID according to Stewart’s approach.

Thirteen patients (12%) had a mixed disorder characterized by a normal pH 7.41 (7.40–7.42) with a lower SID and a lower PaCO_2_ compared with patients without any acid-base disorder.

Eight (8%) patients did not have any acid-base disorder.

The sodium, potassium and chloride concentrations were not different among groups and creatinine levels were within normal range.

### 3.3. Hypoxemia and Acid-Base Disturbance

The whole population was divided according to the median PaO_2_/FiO_2_ value of 199 (Table 4). The median PaO_2_/FiO_2_ were 140 (115–164) in the severe hypoxemic patients and 250 (222–300) in the moderate hypoxemic patients. The respiratory rate and applied PEEP were not different. No relationship between the oxygenation and the respiratory rate was found (*p* = 0.600, R^2^ = 0.02).

The pH was similar between the groups (7.44 (7.43–7.46) vs. 7.44 (7.42–7.46)). Using the Henderson–Hasselbalch approach, severe hypoxemic and moderate hypoxemic group presented similar frequency of respiratory alkalosis, metabolic alkalosis due to aSID and other metabolic alkalosis: 22 (41%) versus 20 (40%), 4 (9%) versus 10 (20%) and 10 (18%) versus 10 (20%). 

## 4. Discussion

The main findings of this observational study in COVID-19 patients with ARDS can be summarized as follows: (1) up to forty percent of the patients presented respiratory alkalosis, (2) up to thirty percent of the patients presented metabolic alkalosis, using both the Henderson–Hasselbalch and Stewart approach, (3) Stewart’s method allows us to classify 12% of the patients with a mixed disorder not detected by the traditional method and (4) the impairment of oxygenation did not affect the acid-base disorders.

A normal acid-base homeostasis is fundamental to guarantee normal physiology and cell function. The presence of any acid-base disorders is associated with higher risk for a worse outcome [19,20]. In non-COVID-19 critically ill patients the most frequently reported acid-base derangement is the acidemia, ranging between 14% and 42%. In a prospective observational study enrolling more than 2500 critically ill patients, 8% had acidemia with an associated intensive care mortality of 57% [8].

The acid-base alterations are generated by several conditions such as respiratory failure, shock, renal and hepatic failure [11]. The severe acid-base derangements are potentially life-threating conditions; thus, a precocious and accurate identification is necessary to improve the outcome [19].

According to the physiological approach the acid-base status depends on the proton concentration (i.e., the pH), on the bicarbonate concentrations [HCO3^-^] and on carbon dioxide (PCO_2_) [11,21]. Acidemia and alkalemia are defined as the accumulation/increase or loss of proton into or from the plasma resulting in a lower or higher pH in absence of any compensatory response. These conditions arise from a respiratory or metabolic alteration [11,12]. In addition, a combination of single derangements in the respiratory or metabolic function can generate a mixed acid-base disorder [11].

The clinical consequences of COVID-19 could range from an asymptomatic condition to a severe disease requiring hospital admission in up to 50% with an associated mortality between 40% and 80% [22,23]. Critically ill patients more often have pneumonia with hypoxemia but can also present other organ dysfunctions such as a cardiovascular, renal and liver failure [6,24]. Thus, these patients, due to several different clinical failures, can present a wide spectrum of acid-base disorders. However, at the present time, only a few studies are available regarding the early assessment of the acid-base disorders in COVID-19 patients [9,25]. The presence of respiratory alkalosis has been detected in between 29.0% and 30.3% [9,25].

In the present study, enrolling 105 COVID-19 patients, 40% of these presented a respiratory alkalosis. Eighty-four percent received a CPAP support, while 16% received non-invasive ventilation. In non-COVID-19 patients the respiratory alkalosis or hypocapnic alkalosis is related to the hyperventilation (increase in respiratory and or tidal volume) associated with the hypoxemia in presence of a pulmonary or central nervous system disease [26]. Wu et al. reported that COVID-19 patients with respiratory alkalosis within the first day from hospital admission had higher rates of underlying diseases and inflammatory markers but similar extension of the disease at lung CT compared to patients without respiratory alkalosis [25]. Similarly, Chen et al., in a cohort of 799 patients, found that the arterial CO_2_ was significantly lower in the patients who died compared to the survivors [27].

Unfortunately, in the present study the tidal volume during CPAP could not be measured. However, the respiratory rate was not different among the acid-base groups and was not related to the PaCO_2_ (*p* = 0.205, R^2^ = 0.01); thus, we hypothesized that the greater minute ventilation was due to the higher tidal volume. In addition, the respiratory rate was not related to the hypoxia and the oxygenation was similar among the groups confirming that, contrary to non-COVID-19 patients with acute respiratory failure, in COVID-19 patients the minute ventilation was not related to the amount of hypoxia [28]. This lower response to the hypoxemia in COVID-19 could be related to the several effects of the virus on the central nervous system and in the lung [28,29], but remains to be elucidated.

The second more frequent acid-base disorder was the metabolic alkalosis with a reported rate similar to those previously showed by Alfano et al. in COVID-19 patients analyzed within the first 48 hours from the hospital admission (31% and 33%, respectively) [9]. In non-COVID-19 the loss of gastric fluid and the use of diuretics account for the majority of cases of metabolic alkalosis [11]. In our population, we can exclude the presence of vomiting or diuretics and the main hypothesis could be the simultaneous presence of fever with previous dehydration due to the long stay at home before hospital admission and the possible activation of the Renin Angiotensin system due to SARS-CoV-2 infection [30]. 

A minority of the patients presented respiratory acidosis with just a slight increase in the arterial PCO_2_; this was related to the early application of the non-invasive ventilation which was able to guarantee an adequate minute ventilation [31].

No patients developed metabolic acidosis because all the patients presented an adequate hemodynamic, without any lactate accumulation or acute renal failure [32].

However, the physiological approach has been questioned because it ignores, according to the principles of physical chemistry the role of water dissociation as a determinant of the pH [13,33]. Taking into account this principle, Stewart suggested a mathematical approach which showed that pH is determined by only three independent variables: the SID, carbon dioxide and the concentration of the weak acids (protein and phosphate). Stewart’s method, by computing the components of the acid-base disorders individually, offers a better understanding of the pathogenesis; in fact, a respiratory acidosis/alkalosis is generated by an increase or decrease in the carbon dioxide, while a metabolic acidosis/alkalosis by a decrease or increase in the SID or in an increase or decrease in the weak acid concentration [13].

In the present study, the SID was computed considering the difference between only the most present anions and cations as sodium, potassium, chloride and lactate. According to this method, 43% and 33% of patients presented a respiratory and metabolic alkalosis, respectively, similar to those obtained by the physiological approach. 

Patients with other metabolic alkalosis presented median SID values within normal range, suggesting the alkalemia was mainly generated by the effect of weak acid, in particular by the albumin and by phosphate. Thus, a decrease in albumin in COVID-19 patients, probably due to a not adequate nutrition or to an increased catabolism, caused a reduction in the weak acids with an alkalinizing effect.

Similarly, 13 patients (12%) had a mixed acid-base disorder characterized by a normal pH with a reduction in SID and a normal or decreased PaCO_2_, then we can hypothesize when PaCO_2_ is within normal range that the normal pH was generated by the combined effect of the strong ions and of the weak acids. 

Moreover, in our population the mixed alkalosis, identified using the Henderson–Hasselbalch approach, can be detected by Stewart’s approach in a subgroup of patients with respiratory alkalosis and with an associated alkalinizing effect of weak acids.

Interestingly, regarding the possible contribution of the hypoxemia in the acid-base disorders, we did not find any effects on either respiratory or metabolic alterations. This could be explained by an adequate oxygen delivery to the organs guaranteed by the non-invasive respiratory support and by the absence of a hemodynamic failure.

### Limitations

Possible limitations of the present study are the retrospective nature, the absence of any data on the patients at hospital admission in the emergency department and the absence of the albumin and phosphate values to calculate the effective SID.

## 5. Conclusions

In conclusion, COVID-19 patients with ARDS treated with non-invasive respiratory support within the first 48 h mainly presented respiratory or metabolic alkalosis. Thus, based on this data, strictly acid-base monitoring should be necessary during the first days of hospital admission in order both to prevent further clinical derangements and to provide an adequate assistance.

## Figures and Tables

**Table 1:** Baseline characteristics of the study population at emergency department admission.

	Number = 104
Age, years	58 (52–64)
Male gender, % (n)	73 (77)
Weight, kg	83 (72–97)
BMI, kg/m^2^	28 (25–33)
Time from symptoms onset to hospital admission, days	6 (4–8)
Time from hospital admission to respiratory support start, days	1 (0–3)
Arterial pH	7.45 (7.43–7.48)
PaCO_2_, mmHg	31.8 (28.4–34.2)
PaO_2_, mmHg	63.0 (55.7–74.0)
PaO_2_/FiO_2_	264 (204–301)
White blood cell count, cells/μL	6600 (5200–8780)
Haemoglobin, g/dL	14.4 (13.1–15.4)
Platelets, cells/μL	198 (152–241)
INR	1.14 (1.08–1.22)
GOT, U/L	55 (41–76)
GPT, U/L	46 (31–71)
Total bilirubin, mg/dL	0.6 (0.4–1.0)
Creatinine, mg/dL	0.8 (0.7–1.0)
LDH, mg/dL	382 (292–472)
D-dimer, ng/mL	300 (226–394)
SOFA score	2 (2–3)

Data are presented as median [IQR]. BMI: body mass index; PaCO_2_: arterial carbon dioxide partial pressure; PaO_2_: arterial oxygen partial pressure; INR: international normalized ratio; GOT: glutamic oxaloacetic transaminase; GTP: glutamic pyruvic transaminase; LDH: L-lactate dehydrogenase; SOFA: sequential organ failure assessment.

**Table 2:** Comparison among groups classified according to Henderson–Hasselbalch approach.

	Study Population	No Acid-Base Disorder	Respiratory Alkalosis	Metabolic Alkalosis	Respiratory Acidosis	Mixed Alkalosis	*p*
Number (%)	104 (100)	14 (13)	42 (40)	34 (32)	5 (5)	9 (10)	-
Age, years	60 (53–69)	59 (52–71)	61 (55–68)	59 (52–66)	58 (57–69)	63 (59–70)	0.824
Female gender, n (%)	28 (27)	21 (3)	17 (7)	41 (14)	100 (5)	13 (1)	0.845
BMI, kg/m^2^	28 (25–33)	25 (25–28)	28 (25–31)	28 (26–34)	30 (30–30)	30 (26–36)	0.804
Time from symptoms to ED, days	6 (4–8)	6 (4–7)	5 (4–8)	6 (3–8)	7 (5–7)	6 (5–8)	0.989
Respiratory rate, bpm	19 (17–24)	20 (17–24)	20 (17–23)	18 (16–22)	18 (16–20)	20 (18–24)	0.791
FiO_2_	70 (60–70)	70 (60–80)	70 (60–80)	65 (60–70)	70 (60–75)	60 (60–70)	0.286
PEEP, cmH_2_O	8 (7.5–10)	10 (8–10)	7.5 (7.5–10)	8 (7.5–10)	7.5 (7.5–10)	7.5 (7.5–8)	0.220
Borg Score	0 (0–0)	0 (0–0)	0 (0–1)	0 (0–0)	0 (0–0)	0 (0–0)	0.203
WOB Score	1 (1–2)	1 (1–2)	1 (1–2)	1 (1–2)	1 (1–4)	2 (1–3)	0.684
Arterial pH	7.44 (7.43–7.46)	7.40 (7.39–7.42)	7.44 (7.44–7.46)	7.45 (7.44–7.46)	7.36 (7.36–7.36)	7.48 (7.48–7.49)	<0.001
PaCO_2_, mmHg	38 (35–41)	40 (39–42)	35 (33–36)	41 (40–44)	48 (44–51)	36 (35–37)	<0.001
PaO_2_, mmHg	123 (92–155)	139 (101–180)	125 (94–177)	108 (92–150)	98 (74–149)	130 (108–146)	0.762
PaO_2_/FiO_2_	199 (139–246)	212 (173–227)	199 (139–261)	167 (140–234)	163 (124–212)	221 (180–246)	0.780
HCO3^−^, mMol/L	25.8 (24.1–27.4)	25.2 (23.8–26.0)	24.2 (22.9–25.0)	28.0 (27.1–29.7)	27 (24.5–28)	27.0 (26.1–27.4)	<0.001
BE, mMol/L	1.6 (0.1–3.6)	1.2 (−0.6–1.7)	0.2 (−1.0–0.8)	4.1 (2.9–6.1)	1.3 (−0.8–3.6)	3.8 (2.1–4.1)	<0.001
Apparent SID, mEq/L	36.6 (34.9–38.2)	37.8 (36.8–38.8)	35.8 (33.9–37.2)	37.6 (36.6–38.9)	35.6 (35.0–36.5)	36.2 (34.9–37.4)	0.004
Sodium, mEq/L	136 (134–138)	138 (135–139)	136 (134–139)	136 (135–138)	137 (133–137)	135 (133–137)	0.333
Potassium, mEq/L	4.1 (3.8–4.3)	4.1 (3.8–4.4)	4.0 (3.8–4.4)	4.1 (3.8–4.3)	4.1 (4.0–4.5)	3.8 (3.6–4.0)	0.270
Cloride, mEq/L	103 (100–105)	102 (100–105)	104 (101–105)	102 (100–104)	100 (99–102)	102 (100–103)	0.151
Lactates, mMol/L	1.3 (1.0–1.7)	1.3 (1.0–1.8)	1.4 (1.2–1.7)	1.2 (1.0–1.4)	1.5 (1.4–2.5)	1.3 (1.0–1.4)	0.075
Anion Gap, mEq/L	8.1 (6.3–10.0)	9.9 (7.9–10.8)	9.0 (8.0–10.7)	6.6 (5.5–7.9)	7.7 (6.0–8.8)	6.9 (5.9–8.0)	<0.001
Creatinine, mg/dL	0.9 (0.7–1.1)	0.8 (0.7–0.95)	0.9 (0.8–1.1)	0.7 (0.6–0.9)	1.0 (0.9–1.2)	1.0 (0.9–1.2)	0.007
Ventilation type, n (%)							0.290
CPAP	84 (87)	100 (14)	81 (34)	85 (29)	80 (4)	67 (6)	
NIV	16 (17)	0 (0)	19 (8)	15 (5)	20 (1)	33 (3)	
Endotracheal Intubation, n (%)	19 (20)	26 (4)	19 (8)	9 (3)	2 (40)	33 (3)	0.223
Mortality, n (%)	14 (13)	2 (14)	5 (2)	15 (5)	40 (2)	33 (3)	0.065

Continuous variables are compared using One-Way ANOVA or Kruskal–Wallis Test, as appropriate; categorical variables are compared using χ^2^ test. Data are presented as median [IQR]. BMI: body mass index; FiO_2_: inspired oxygen fraction; ED: emergency department; PEEP: positive end-expiratory pressure; WOB: work of breathing; PaCO_2_: arterial carbon dioxide partial pressure; PaO_2_: arterial oxygen partial pressure; HCO_3_^−^: arterial bicarbonate concentration; BE: base excess; SID: strong ion difference; CPAP: continuous positive airway pressure; NIV: non-invasive ventilation.

**Table 3:** Comparison among groups classified according to Stewart approach.

	Study Population	No Acid-Base Disorder	Respiratory Alkalosis	Metabolic Alkalosis due to aSID	Other Alkalosis	Respiratory Acidosis	Mixed Disorder	*p*
Number (%)	104 (100)	8 (8)	45 (43)	20 (19)	14 (14)	4 (4)	13 (13)	-
Age, years	60 (53–69)	58 (53–64)	60 (53–68)	61 (50–70)	60 (54–68)	64 (58–69)	62 (58–65)	0.943
Female gender, n (%)	28 (27)	1 (12)	8 (18)	11 (52)	6 (43)	25 (1)	8 (1)	0.654
BMI, kg/m^2^	28 (25–33)	25 (25–28)	28 (25–31)	28 (26–34)	28 (26–33)	30 (26–36)	30 (30–30)	0.734
Time from symptoms to ED, days	6 (4–8)	6 (4–7)	5 (4–8)	4 (4–6)	6 (4–8)	6 (5–8)	7 (5–7)	0.783
Respiratory rate, bpm	19 (17–24)	19 (17–26)	19 (18–22)	18 (16–22)	18 (16–22)	19 (18–22)	19 (16–24)	0.668
FiO_2_	70 (60–70)	70 (60–70)	70 (60–70)	70 (60–70)	60 (60–70)	75 (70–75)	60 (60–70)	0.532
PEEP, cmH_2_O	8 (7.5–10)	10 (7.5–10)	7.5 (7.5–10)	8 (7.5–10)	8 (7.5–10)	10 (9–10)	10 (7.5–10)	0.141
Borg Score	0 (0–0)	0 (0–1)	0 (0–1)	0 (0–0)	0 (0–0)	0 (0–0)	0 (0–1)	0.145
WOB Score	1 (1–2)	1 (1–3)	1 (1–2)	1 (1–2)	1 (1–2)	1 (1–2)	1 (1–2)	0.971
Arterial pH	7.44 (7.43–7.46)	7.41 (7.40–7.42)	7.45 (7.44–7.48)	7.45 (7.44–7.46)	7.44 (7.45–7.46)	7.36 (7.36–7.36)	7.41 (7.40–7.42)	<0.001
PaCO_2_, mmHg	38 (35–41)	41 (40–42)	35 (33–36)	40 (39–42)	39 (38–42)	48 (45–54)	37 (34–43)	<0.001
PaO_2_, mmHg	123 (92–155)	96 (81–190)	120 (93–165)	122 (90–152)	126 (97–145)	124 (92–195)	138 (88–145)	0.983
PaO_2_/FiO_2_	199 (139–246)	221 (118–298)	199 (138–276)	173 (150–230)	168 (146–240)	188 (148–278)	198 (143–234)	0.923
HCO3^−^, mMol/L	25.8 (24.1–27.4)	25.6 (24.2–25.9)	24.7 (23.6–25.6)	27.4 (26.4–29.6)	28.0 (26.9–29.4)	26.2 (24.4–28.5)	23.4 (22.0–27.0)	0.001
BE, mMol/L	1.6 (0.1–3.6)	1.6 (0.9–1.9)	0.5 (−0.6–1.4)	3.4 (2.4–6.0)	3.6 (2.5–6.1)	0.25 (−1.7–2.8)	1.4 (−2–3.6)	0.001
Apparent SID, mEq/L	36.6 (34.9–38.2)	38.6 (38.0–39.1)	38.9 (38.4–39.4)	42.2 (42.1–43.0)	40.0 (38.6–40.5)	38.8 (38.0–39.6)	35.6 (34.4–37.4)	0.034
Sodium, mEq/L	136 (134–138)	139 (136–139)	136 (133–139)	137 (135–138)	137 (135–138)	137 (136–138)	137 (134–139)	0.437
Potassium, mEq/L	4.1 (3.8–4.3)	4.2 (4.0–4.4)	4.0 (3.8–4.3)	4.0 (3.7–4.2)	4.1 (3.7–4.3)	4.3 (4.1–4.5)	4.3 (4.0–4.4)	0.198
Cloride, mEq/L	103 (100–105)	102 (99–103)	103 (101–105)	101 (100–104)	101 (100–104)	102 (100–104)	103 (102–106)	0.209
Lactates, mMol/L	1.3 (1.0–1.7)	1.1 (0.9–1.3)	1.3 (1.1–1.6)	1.1 (1.0–1.4)	1.1 (1.1–1.4)	2.0 (1.4–2.6)	1.7 (1.2–1.9)	0.087
Creatinine, mg/dL	0.9 (0.7–1.1)	0.8 (0.7–0.95)	0.9 (0.8–1.1)	1.0 (1.0–1.2)	1.1 (0.9–1.1)	1.0 (0.9–1.2)	0.7 (0.6–0.9)	0.004
Ventilation type, n (%)								0.489
CPAP	84 (87)	8 (100)	35 (78)	10 (50)	10 (71)	3 (75)	12 (92)	
NIV	16 (17)	0 (0)	10 (22)	10 (50)	4 (29)	1 (25)	1 (8)	
Endotracheal Intubation, n (%)	19 (20)	4 (50)	9 (20)	2 (10)	2 (14)	2 (50)	2 (15)	0.132
Mortality, n (%)	13 (14)	1 (12)	5 (11)	4 (20)	1 (7)	2 (50)	1 (8)	0.136

Continuous variables are compared using One-Way ANOVA or Kruskal–Wallis Test, as appropriate; categorical variables are compared using χ^2^ test. Data are presented as median [IQR]. AB: acid-base; BMI: body mass index; FiO_2_: inspired oxygen fraction; PEEP: positive end-expiratory pressure; ED: emergency department; WOB: work of breathing; PaCO_2_: arterial carbon dioxide partial pressure; PaO_2_: arterial oxygen partial pressure; HCO_3_^−^: arterial bicarbonate concentration; BE: base excess; SID: strong ion difference; CPAP: continuous positive airway pressure; NIV: non-invasive ventilation.

**Table 4:** Comparison between groups classified according to the median value of PaO_2_/FiO_2_.

	Severe Hypoxemia	Moderate Hypoxemia	*p*
Number, (%)	51 (51)	53 (49)	-
Age, years	62 (54–68)	59 (53–70)	0.941
BMI, kg/m^2^	28 (26–33)	28 (25–33)	0.366
Time from symptoms to ED, days	6 (4–7)	5 (4–9)	0.692
Respiratory rate, bpm	19 (17–24)	19 (16–22)	0.520
FiO_2_	60 (60–70)	70 (60–70)	0.028
PEEP, cmH_2_O	8 (7.5–10)	7.5 (7.5–10)	0.380
Borg Score	0 (0–0)	0 (0–0)	0.410
WOB Score	1 (1–2)	1 (1–2)	0.126
Arterial pH	7.44 (7.43–7.46)	7.44 (7.42–7.46)	0.829
PaCO_2_, mmHg	38 (35–42)	38 (34–41)	0.185
PaO_2_, mmHg	92 (78–99)	155 (140–204)	<0.001
PaO_2_/FiO_2_	139 (115–162)	246 (221–398)	<0.001
HCO3^−^, mMol/L	25.8 (24.4–27.4)	25.8 (23.6–27.4)	0.320
BE, mMol/L	1.6 (0.4–3.6)	1.4 (−0.68–3.8)	0.528
Apparent SID, mEq/L	36 (35–38)	37 (35–38)	0.761
Sodium, mEq/L	136 (135–139)	137 (133–138)	0.189
Potassium, mEq/L	4.1 (3.8–4.4)	4.1 (3.8–4.2)	0.199
Cloride, mEq/L	103 (101–105)	102 (100–104)	0.062
Lactates, mMol/L	1.3 (1.1–1.6)	1.3 (1.0–1.7)	0.780
Creatinine, mg/dL	0.9 (0.7–1.1)	0.8 (0.7–1.0)	0.448
Endotracheal intubation, n (%)	13 (25)	7 (13)	0.271
Acid-base disorders, n (%)			0.491
No Acid-base disorder	5 (10)	9 (18)	
Respiratory Acidosis	3 (5)	2 (3)	
Respiratory Alkalosis	20 (40)	22 (41)	
Metabolic Alkalosis due to aSID	10 (20)	4 (9)	
Other Metabolic Alkalosis	10 (20)	10 (18)	
Mixed Alkalosis	3 (5)	6 (11)	

Continuous variables are compared using Student’s *t*-test or Wilcoxon–Mann–Whitney Test, as appropriate; categorical variables are compared using X^2^ test. Data are presented as median [IQR]. ED emergency Department; BMI: body mass index; FiO_2_: inspired oxygen fraction; PEEP: positive end-expiratory pressure; WOB: work of breathing; PaCO_2_: arterial carbon dioxide partial pressure; PaO_2_: arterial oxygen partial pressure; HCO_3_^−^: arterial bicarbonate concentration; BE: base excess; SID: strong ion difference.

## Data Availability

Not applicable.
